# The Mediterranean Diet, the DASH Diet, and the MIND Diet in Relation to Sleep Duration and Quality: A Narrative Review

**DOI:** 10.3390/nu16234191

**Published:** 2024-12-04

**Authors:** Dorota Różańska

**Affiliations:** Department of Dietetics and Bromatology, Wroclaw Medical University, 50-556 Wroclaw, Poland; dorota.rozanska@umw.edu.pl

**Keywords:** sleep quality, sleep duration, sleep hygiene, insomnia, Mediterranean diet, DASH diet, MIND diet

## Abstract

In 2022, healthy sleep was included as part of Life’s Essential 8, which are a cluster of fundamental factors for cardiovascular health. In many studies, sleep duration and/or quality have also been found to be related to human health. The purpose of this narrative review was to present the relationship between the Mediterranean diet, the DASH (Dietary Approaches to Stop Hypertension) diet, and the MIND (Mediterranean–DASH Intervention for Neurodegenerative Delay) diet and sleep quality and duration in different populations. A literature search was conducted based on the phrases “Mediterranean diet”, “DASH diet”, and “MIND diet” appearing together with “sleep” or “insomnia” in papers’ titles or abstracts. Studies on obstructive sleep apnea or shift work were excluded. The electronic databases were searched via EBSCOhost. Main results: The vast majority of studies showed that there was a beneficial association between the three diets discussed in this review and sleep duration and/or quality; however, most of these results were obtained in cross-sectional analyses. There were only a few studies in which an association between sleep parameters and these diets was not observed. Direction for future studies: Taking into account the significant role of adequate sleep quality in various diseases, even in all-cause mortality, the implementation of randomized controlled trials and prospective cohort studies seems to be necessary to provide further evidence that would allow these associations to be confirmed. In conclusion, the results obtained so far in various populations strongly suggest that healthy dietary patterns abundant in plant foods, are associated with better sleep quality, as well as with a more adequate length of sleep.

## 1. Introduction

In 2022, healthy sleep, along with a healthy diet, physical activity, avoidance of nicotine, healthy weight, healthy levels of blood lipids and blood glucose, and healthy blood pressure, was included as part of Life’s Essential 8, which constitutes a cluster of fundamental factors in optimizing and preserving cardiovascular (CV) health [1]. It is important to underline that healthy sleep not only refers to an adequate sleep duration but also other elements, such as its timing, regularity, efficiency, satisfaction, and impact on daytime alertness [1]. In recent years, the role of sleep quality as a factor that can influence human health has been shown by many authors [2,3,4,5,6,7,8,9,10]. A meta-analysis published by Lian et al. [2] clearly demonstrated that different aspects of sleep quality should be considered to have an association with health. It was found that not only the general quality of sleep had a significant positive association with metabolic syndrome, but also particular problems, such as difficulty falling asleep, difficulty maintaining sleep, and sleep inefficiency were associated with metabolic syndrome. Taking into account sleep duration, too short, as well as too long, of a sleep duration may adversely affect human health. Kim et al. [3] conducted a study among 133,608 subjects and showed that less than 6 h of sleep in comparison with 6–8 h was associated with a higher odds of metabolic syndrome and an increased waist circumference among men and with a higher odds of an increased waist circumference and fasting glucose among women. On the other hand, more than 10 h of sleep in comparison to 6–8 h was associated with a higher odds of metabolic syndrome and raised triglycerides in both men and women, as well as with a higher odds of increased waist circumference and fasting glucose levels and reduced HDL-cholesterol levels in women [3]. A higher risk of nonalcoholic fatty liver disease (NAFLD), which is commonly linked with metabolic syndrome, was also associated with an inadequate length of sleep [4,5]. Other authors, in a meta-analysis based on data from 28 articles involving 95,259 older people overall, found a statistically significant relationship between prolonged sleep time and an increased risk of all-cause mortality in both women and men. However, reduced sleep time was significantly associated with an increased risk of all-cause mortality only among men and not among women [6]. Gao et al. [7], based on a review of 85 meta-analyses, concluded that there is highly suggestive evidence that long sleep time is associated with a higher risk of all-cause mortality. Suggestive evidence that a prolonged sleep time is also associated with a higher risk of stroke and mortality due to stroke, dyslipidemia, and mortality due to coronary heart disease (CHD) was also found. On the other hand, these authors also observed noticeable evidence that a short sleep duration is related to a greater risk of overweight and/or obesity, while poor sleep quality is related to a lower risk of diabetes mellitus and gestational diabetes mellitus. Furthermore, Lu et al. [8] showed that a higher risk of type 2 diabetes was caused by too short as well as too long a sleep duration. It is also important to highlight that an inadequate sleep time may increase the risk of depression [9] and, on the other hand, that improving the quality of sleep may be beneficial for improving mental health [10].

What is also interesting is that sleep duration may be a factor that significantly modifies the association between the risk of cardiovascular disease (CVD) and diet. Results from the ATTICA Cohort Study showed that higher adherence to the Mediterranean diet (MD) was inversely associated with CVD risk, but there was also a significant interaction between length of sleep and the MD. Furthermore, in a subgroup analysis, the beneficial influence of the MD on CVD risk was noted only in the group of participants with a proper sleep duration (≥7 h/day). Moreover, a 70% reduced 20-year CVD risk was observed in the group of participants who had high adherence to the MD and an adequate sleep duration in comparison with the group with a low adherence to the MD and limited hours of sleep (<7 h/day) [11]. Furthermore, according to the National Health and Nutrition Examination Survey (NHANES), lower adherence to the MD and sleep disorders synergistically increased long-term total and CV mortality [12]. Adherence to the MD was calculated based on the alternative Mediterranean diet (aMed) index and divided into three groups: above median, median, and below median. It was found that lower compliance with the MD significantly increased the risk of total and CV mortality, by 42% and 13%, respectively (in the below vs. above median groups). Furthermore, sleep disorders also significantly increased the risk of total and CV mortality, by 42% and 61%, respectively. A further analysis showed the joint effect of lower values in the aMed index and sleep disorders. The greatest risk of total and CV mortality was observed in those with lower values in the aMed index along with a sleep disorder. A significant interaction between the aMed index and sleep disorders that affects CV mortality was found. Moreover, in the groups of participants who had a low or medium aMed index along with a sleep disorder, a higher cumulative risk of total mortality and cumulative CV mortality during the follow-up was observed [12].

The above-mentioned observations from different studies give a lot of arguments for treating healthy sleep as one of the factors that is associated with human health. Therefore, it is worth thinking about possible actions that may be helpful in ensuring better sleep quality. 

The purpose of this narrative review was to present the relationships between the Mediterranean diet, the DASH (Dietary Approaches to Stop Hypertension) diet, and the MIND (Mediterranean–DASH Intervention for Neurodegenerative Delay) diet and sleep quality and duration in different populations.

## 2. Methods

This narrative review focuses on various studies that assessed the associations between the Mediterranean diet, the DASH diet, and the MIND diet and sleep quality and/or its duration in different populations. The following electronic databases via EBSCOhost were searched: MEDLINE Ultimate, MEDLINE, CINAHL Ultimate, Academic Search Ultimate, and Health Source: Nursing/Academic Edition. The literature search was conducted in August and September 2024 in three steps. First, the literature search was based on the words “Mediterranean diet”, “DASH diet”, and “MIND diet” appearing together with “sleep” or “insomnia” in the titles or abstracts. Next, studies on obstructive sleep apnea or shift work were excluded from this review. The inclusion criteria were English language and full-text availability. Cross-sectional, prospective, case–control, and randomized controlled trials (RCTs) were included. Different populations were taken into account, from children and adolescents through adults and up to the elderly. Adherence to the Mediterranean diet, DASH diet, or MIND diet in the selected studies was assessed using a priori methods. Finally, abstracts and full texts were read to confirm that the chosen articles aligned with the purpose of the review. As this is a narrative review, there was no need to document and show the literature search on specific platforms [13].

## 3. The Mediterranean Diet

### 3.1. Characteristics of the Mediterranean Diet and Its Association with Health

The Mediterranean diet (MD) has been widely known for many years because of its benefits for human health. The MD was defined thanks to the Seven Countries Study, in which an association between this dietary pattern and CHD was observed [14,15]. It is worth underlining that the MD was not a diet created by scientists, physicians, or dieticians, but it describes the traditional food patterns common to Crete, much of the rest of Greece, and southern Italy during the early 1960s. Locally grown and minimally processed foods, as well as seasonal products, constitute the basis of the traditional MD. This diet is associated with a high intake of fruits, vegetables, minimally refined breads, and other cereals. The content of other plant foods, such as potatoes, beans, nuts, and seeds, is also high in the traditional MD. The traditional MD is also characterized by a high consumption of olive oil. It contains moderate amounts of dairy products, low to moderate amounts of fish and poultry, and low amounts of red meat. The consumption of eggs is limited to up to four per week. In the traditional MD, fresh fruits are also consumed as a usual dessert, with sweets containing sugars or honey a few times per week. Wine is consumed in moderation, with meals [15]. Over the years, numerous studies have been conducted on the relationship between the MD and various health outcomes. In one of the meta-analyses of randomized clinical trials, the MD was found to be inversely associated with total CVD and total myocardial infarction incidence. In a meta-analysis of prospective cohort studies, the MD was inversely associated with total CVD mortality, CHD incidence and mortality, stroke incidence and mortality, and myocardial infarction incidence [16]. Another meta-analysis was based on RCTs, case–control, and cohort studies and included 3,202,496 participants overall. The analysis showed that the MD decreased the risk of cancer mortality and total mortality among cancer survivors, and also decreased the risk of some cancers, such as head and neck, respiratory, breast, colorectal, gastric, bladder, and liver [17]. Kawaguchi et al. [18] showed that the MD improved hepatic steatosis and insulin resistance in patients with NAFLD. Moreover, the MD was found to be associated with a lower risk of mild cognitive impairment [19], Alzheimer’s disease [19,20], and Parkinson’s disease [20]. The MD also had a favorable effect in decreasing the risk of depression [21,22]. It is worth noting that the MD was inscribed by UNESCO on the Representative List of the Intangible Cultural Heritage of Humanity [15,23].

### 3.2. The Mediterranean Diet and Its Association with Sleep—A Review

Recently, various studies conducted in different populations have focused on the MD and its association with sleep quality. One of them was the longitudinal Multi-Ethnic Study of Atherosclerosis (MESA), in which higher adherence to the MD was found to be associated with adequate sleep duration and fewer insomnia symptoms. It was observed that participants who currently reported a moderate–high alternate Mediterranean diet (aMed) score were significantly more likely to sleep 6–7 h/night (vs. <6 h) and less likely to declare insomnia symptoms occurring with short sleep (vs. no insomnia or short sleep alone) than those who had a low aMed score value. However, there was no relationship between sleep duration or insomnia symptoms when an increase in the aMed score over the preceding 10 years was taken into account. On the other hand, compared with participants with a decreasing aMed score, participants with an unchanging aMed score declared significantly fewer symptoms of insomnia [24]. Another study in which sleep duration and the MD were assessed was conducted among inhabitants of Costa Rica. The authors concluded that short and inconsistent sleep durations may affect the dietary patterns of the study population. They found that women who had limited sleep duration on weekdays (<7 h/night) were more likely to have lower aMed score values compared with those who had the recommended sleep duration (7–9 h/night). However, in the group of men, no significant association between sleep duration on weekdays and aMed score values was noted. Moreover, an inconsistent weekday–weekend sleep duration (more than a 1 h difference) was observed to be associated with lower compatibility with the MD. However, no association between the frequency of napping and the aMed score was found [25]. In a study conducted among adults in Ankara, the total sleep duration among participants with low MD adherence was significantly lower (7.5 ± 1.5 h) than among those with moderate and high MD adherence (8.3 ± 1.4 and 8.2 ± 1.3 h, respectively). There was also a significant difference in the percentages of participants who woke up <9:00, between 9:00 and 10:00, and ≥10:00 across the three MD adherence groups. In the low MD adherence group, 40.4% of participants woke up <9:00 and 28.5% woke up ≥10:00, while in the high MD adherence group, these values were 53.2% and 21.5%, respectively [26].

In a prospective cohort study conducted among American women, higher aMed score values at baseline were associated with better sleep quality, higher sleep efficiency, and a lower number of sleep disturbances at the 1-year follow-up. Sleep quality was assessed with the Pittsburgh Sleep Quality Index (PSQI), which is the most common self-report method used to measure habitual sleep quality [27]. Studies conducted in Italy also showed a relationship between higher compatibility with the MD and better quality of sleep [28,29]. However, in an additional analysis, Godos et al. [28] observed such results only among normal/overweight participants but not in those who were obese. Similar results were observed in a study conducted among adults from Shanghai, where the prevalence of poor sleep was lower among participants with higher adherence to the MD (4th quartile vs. 1st quartile OR = 0.75; 95% CI 0.64–0.87) [30]. The association between higher adherence to the MD and good sleep quality, even after controlling for potential confounders, was also observed by Shiraseb et al. [31] among overweight and obese women from a health center in Tehran. Zaidalkilani et al. [32] conducted a study among women in Jordan and showed that adherence to the MD was significantly associated with better sleep as well as with fewer symptoms of insomnia. The relationship between Mediterranean dietary patterns and better sleep quality was also reported by Şahin-Bodur et al. [26] and Bakırhan et al. [33]. 

There are also some studies conducted among the elderly population in which higher compatibility with the MD was correlated with better sleep quality [34,35,36] or with a decreased risk of changes in sleep duration [35]. However, Mamalaki et al. [36] did not observe an association between sleep duration and MD adherence. On the other hand, in a study conducted among 6–7-year-old Italian children, adherence to the MD was associated with the number of hours of sleep. Children with good sleep duration compared to those with poor sleep duration (≥10 vs. <10 h) were less likely to have poor-to-moderate adherence to the MD [37]. There is also evidence from studies conducted among adolescents about the relationship between the MD and sleep quality. Poor sleep patterns, including not only problems such as difficulties in awakening and falling asleep but also the presence of nighttime nightmares and morning tiredness, were associated with lower adherence to the MD among schoolchildren from Chile. The MD was also associated with their wake-up time and bedtime [38]. Other authors found that adherence to the MD was inversely associated with insomnia problems among Iranian adolescent girls [39]. The MD was also associated with sleep duration and sleep quality among Italian adolescents. In the groups with medium and high adherence to the MD, sleep duration was adequate. Also, a diet more similar to the MD was associated with daytime sleepiness. Higher drowsiness was observed among those who had lower adherence to the MD [40]. Spanish adolescents who had high adherence to the MD were more likely to meet the sleep recommendations, as well as less likely to declare at least one sleep-related problem (bedtime problems, excessive daytime sleepiness, awakening during the night, regularity and duration of sleep, or sleep-disordered breathing) [41]. Likewise, a relationship between the MD and sleep quality was observed among students [42]. It was also found that good compatibility with the MD was associated with better subjective sleep quality and less sleep latency, sleep disturbance, and daytime dysfunction, as well as with the morningness chronotype. However, there was no association between adherence to the MD and sleep duration, sleep efficiency, or the use of sleep medications [43]. Diets that were not similar to the MD were also associated with higher degrees of insomnia and sleepiness during the day among young women [44]. 

What is also interesting is that higher adherence to the MD was found to be related to better sleep quality throughout the pregnancy course [45]. Moreover, interventions based on the MD during pregnancy were associated with improvements in sleep quality throughout pregnancy. This beneficial influence was observed in a randomized clinical trial, in which pregnant women at high risk for small-for-gestational-age newborns were randomly divided into one of three groups. In the first group, the Mediterranean diet supplemented with extra-virgin olive oil and walnuts was implemented. In the second group, a stress reduction intervention program was implemented. The third group was a control group, with only usual care without any intervention [46].

Despite the many results mentioned above, in which an association between the Mediterranean dietary pattern and sleep quality was observed, not all authors found such a relationship. Verkaar et al. [47] conducted cross-sectional and longitudinal analyses based on data from the Rotterdam Study and did not observe any statistically significant associations between different dietary patterns (MD, among others) and objective and subjective sleep outcomes, such as total sleep time, sleep onset latency, wake after sleep onset, sleep efficiency, and PSQI. No association between the MD and sleep parameters (sleep initiating problems, sleep maintenance problems) was found by van Egmond et al. [48] in a study conducted among older men from Sweden. A summary of the main findings from the research cited above, according to the relationship between the MD and sleep quality, is presented in Table 1.

## 4. The DASH Diet

### 4.1. Characteristics of the DASH Diet and Its Association with Health

The DASH trial (Dietary Approaches to Stop Hypertension) was initially conducted to assess the effectiveness of a specially designed diet in lowering blood pressure. This diet included plenty of fruits, vegetables, and low-fat dairy while minimizing the intake of cholesterol, saturated fatty acids, and total fats. The DASH diet was designed based on different energy levels. In the diets designed for 2100 kcal, the nutrient targets for potassium, magnesium, and calcium were 4700 mg, 500 mg, and 1240 mg per day, respectively. The assumption for fiber and protein intake was 31 g per day and 18% of total energy intake, respectively [49]. In the diet designed for 2100 kcal, there were 7.7, 5.2, and 4.4 servings of grains, fruits and juices, and vegetables, respectively. There were also two servings of low-fat and 0.7 servings of regular-fat dairy products. The DASH diet contained 0.6 and 0.5 servings of poultry and fish, respectively, but also 0.5 servings of red meat (beef, ham, other pork). The DASH diet also included about 0.5 servings of nuts and seeds daily, 0.1 servings of legumes, 2.5 servings of fat, oils, and salad dressings, and 0.5 servings of snack foods and sweets [50]. Both the DASH diet and the MD are recommended as non-pharmacological tools for the treatment of elevated blood pressure and cardiovascular risk reduction, with class I recommendations and level A evidence [51]. Over the years, many studies have shown the beneficial effects of the DASH diet in other health conditions, including reducing total and LDL-cholesterol [52]. It has also been shown that adherence to this diet may have a positive influence on reducing the risk of metabolic syndrome or improving its components [53,54]. The DASH diet was also associated with a reduction in hepatic fibrosis, steatosis, and liver enzyme levels among patients with NAFLD [55] and with a decreased risk of NAFLD [56]. Various studies have shown many other positive effects of the DASH diet, such as lowering the risks of breast cancer [57,58], colorectal, colon, and rectal cancer [59], bladder cancer [60], head and neck cancer [61], and all-cause, CVD, stroke, and cancer mortality [62]. 

### 4.2. The DASH Diet and Its Association with Sleep—A Review

Taking into account that the DASH diet has a beneficial impact on human health, it seems interesting to analyze whether there is also an association between this diet and sleep, but there are not as many studies as there are for the MD. However, some studies published in recent years support the hypothesis that diets more compliant with the principles of the DASH diet may be related to better sleep quality. Daneshzad et al. [63] conducted a RCT among women with type 2 diabetes in which participants were randomly divided into a DASH diet group or a control diet group for 3 months. After this time, in the first group, the length of sleep at night increased significantly (from 6.61 ± 1.09 to 7.53 ± 1.20 h), and the sleep score (PSQI) decreased (from 16.27 ± 5.06 to 8.66 ± 5.80 points). Moreover, after 12 weeks of the intervention, the DASH diet significantly decreased testosterone levels, 2 h postprandial glucose levels, and advanced glycation end products, as well as scores for depression and anxiety [63]. The positive outcome of the DASH diet was also observed by Liang et al. [64] in the National Health and Nutrition Examination Surveys. It was found that a higher DASH diet quality score was inversely associated with poor sleep-related daytime dysfunction in the overall study group, after adjusting for age, sex, and demographic and socio-economic factors. However, no significant relationship was found between sleepiness and sleep disturbance. Huang et al. [30] observed that higher adherence to the DASH diet was associated with a lower prevalence of poor sleep, as assessed with the PSQI. There was about a 30% lower risk of poor sleep prevalence in the fourth quartile vs. the first quartile of the DASH diet score. Another study that may confirm the relationship between sleep and the DASH diet was conducted among overweight and obese women. Higher adherence to the DASH diet was associated with good quality of sleep. A significant relationship between the DASH diet and circadian rhythm was also found [31]. Another reason to consider the DASH diet as a factor that may affect sleep quality was observed by Saharkhiz et al. [65] in a study conducted in a group of young girls. In multivariate multinomial logistic regression analyses, higher compatibility with the DASH diet (third vs. first tertile) was associated with less difficulty with sleep initiation. However, no significant association between the DASH diet score and insomnia, daytime sleepiness, or short sleep duration was found. Other authors found an inverse association between adherence to the DASH diet and insomnia in a group of adolescent girls [66]. Moreover, an inverse association between adherence to the DASH diet and daytime sleepiness was observed in a different study conducted in a group of adolescent girls [67]. 

Karbasi et al. [68] observed interesting findings in a study conducted among breastfeeding mothers and their infants. The authors observed that the DASH diet may have a favorable effect not only for mothers but also for infants whose mothers had a diet similar to the DASH dietary pattern. The benefits of the DASH diet were observed in lower scores for sleep latency and sleep disorders among mothers, as well as in higher sleep efficiency. Among infants whose mothers had higher adherence to the DASH diet compared to those whose mothers had low adherence to this dietary pattern, fewer sleep disorders were observed. The DASH dietary pattern was not associated with a need for sleep medications or daytime dysfunction among the mothers.

On the other hand, in a case–control study conducted among individuals who had recovered from COVID-19, no association was observed between sleep quality and insomnia with this diet [69]. The main findings from the above studies, according to the relationship between the DASH diet and sleep quality, are summarized in Table 2.

## 5. The MIND Diet

### 5.1. Characteristics of the MIND Diet and Its Association with Health

The Mediterranean–DASH Intervention for Neurodegenerative Delay diet (MIND diet) is a combination of the MD and the DASH diet, but with modifications associated with food intake that has been found to be related to the risk of dementia. It has been observed that this dietary pattern has a significant impact on slowing cognitive decline with age. In this diet, ten food groups (green leafy vegetables, other vegetables, nuts, berries, beans, whole grains, seafood, poultry, olive oil, and wine) have been classified as brain healthy food groups, whereas five (red meats, butter and stick margarine, cheese, pastries and sweets, and fried/fast food) have been classified as unhealthy food groups [70]. Many studies have shown that this diet may have positive results in slowing down the progression of cognitive decline and in delaying the development and progression of Parkinson’s disease [71,72,73]. Moreover, the MIND diet was found to be associated, for example, with a lower risk of dementia in middle-aged and older adults [74] and better verbal memory in later life [75]. This diet also had a beneficial association with improving anthropometric parameters and other cardiometabolic outcomes (blood pressure, glycemic control, lipid profile, inflammation, and stroke) [76] and reducing the risk of cardiovascular events [77] and breast cancer [78,79]. Moreover, a relationship was found between the MIND diet and lower risks of depression and anxiety [80]. In a study conducted among 882 participants, the MIND diet was also significantly associated with a decreased risk of total mortality in over 12 years of follow-up [81].

### 5.2. The MIND Diet and Its Association with Sleep—A Review

Despite the fact that various studies on the effect of the MIND diet have been published over many years, data on the association between the MIND diet and sleep quality are very limited. One of the studies that has taken into account this problem was conducted among men. It was found that the MIND diet was associated with a lower risk of poor sleep quality, with an odds ratio for the third vs. first tertile of 0.58 (95% CI 0.34–0.98) in a model adjusted for confounding variables. Moreover, individuals in the third tertile, in comparison with the first tertile of the MIND diet had 42% and 46% lower odds of daytime sleepiness and insomnia in the multivariable-adjusted models, respectively [82]. Another study was also a cross-sectional analysis, but conducted among patients with Parkinson’s disease. Among others, the association between the MIND diet and patient-reported outcomes in Parkinson’s disease was measured. It was found that the MIND diet score had significant reductions in many non-motor symptoms, including those associated with sleep, such as daytime sleepiness, insomnia, and REM sleep behavior disorder. Interestingly, the MIND diet scores were associated with a greater reduction in the severity of constipation, anxiety, fatigue, insomnia, forgetfulness, and sexual dysfunction compared to the MD scores [83]. Table 3 shows a summary of the main findings from the studies cited above according to the relationship between the MIND diet and sleep quality.

## 6. Discussion

The aim of this review was to present the results of studies that analyzed the associations between the MD, the DASH diet, and the MIND diet with sleep quality parameters and sleep duration in different populations. Among these three diets, the greatest amount of research has been conducted on the association between the MD and various sleep parameters; however, most of these studies were cross-sectional studies, with only a few prospective cohort studies or randomized clinical trials. The vast majority of available data showed a favorable relationship between the MD and sleep quality and/or sleep duration in various populations: children, adolescents, adults, the elderly, or pregnant women [24,25,26,27,28,29,30,31,32,33,34,35,37,38,39,40,41,42,44,45,46]. However, not all the results support these observations. For example, Mamalaki et al. [36] and Naja et al. [43] found an association between the MD and sleep quality but not between the MD and sleep duration. Moreover, Verkaar et al. [47] and Egmond et al. [48] did not observe a relationship between the MD and various sleep parameters. 

Although the DASH diet has also been known for many years, there are not as many studies on its association with sleep as there are for the MD. During the literature search, mainly cross-sectional studies were found [30,31,64,65,66,67,68]; only one RCT [63] and one case–control study [69] were found. Among these studies, only three [30,64,69] included both men and women in a study group (among which one involved cases of recovered COVID-19 patients and controls who had no history of COVID-19 [69]). In the remaining six studies [31,63,65,66,67,68], the study group included only girls or women (one study included diabetic patients [63], and one study included breastfeeding mothers and their infants [68]). Most of the results showed that the DASH diet was associated with better sleep quality or a lower prevalence of some sleep-related problems [30,31,63,66,67,68]. However, not all of the results are consistent. Saharkhiz et al. [65] showed that the DASH diet was only associated with fewer problems with sleep initiation, but they did not observe an association with insomnia, daytime sleepiness, or short sleep duration. Liang et al. [64] also did not observe an association between the DASH diet and sleepiness or sleep disturbances; however, there was an association with poor sleep-related daytime dysfunction. In the study conducted by Rostami et al. [66], the DASH diet was associated with insomnia, and in the study conducted by Pahlavani et al. [67], it was associated with daytime sleepiness. On the other hand, the results obtained by Khorasanchi et al. [69] provided no evidence for the hypothesis that the DASH diet is associated with sleep quality or insomnia.

The third diet discussed in this review was the MIND diet, which is the newest of the three, having been designed in 2015 [70]. There are very little data about the association between the MIND diet and sleep; however, this may be a future direction for further research, taking into account the promising results from most studies on the Mediterranean and the DASH diets. 

The three diets discussed in this review share many common features, the most important of which is a high consumption of vegetables and fruits, as well as grains. These diets also pay attention to other plant food consumption, such as legumes, seeds, and nuts. Monounsaturated fatty acids have the largest part in fat intake, while saturated fatty acid intake is limited. However, the total amount of fat in the MD is much higher than in the DASH diet. Meat consumption in these diets is low. The composition of these diets clearly suggests that healthy dietary patterns are associated with better quality of sleep and/or its duration. This hypothesis may be confirmed by one of the analyses conducted as part of the NHANES [84]. In the study, conducted among 19,892 participants, the highest adherence to the dietary pattern named “low whole grains, vegetables, and fruits” (which was defined by low consumption of oils, whole grains, nuts and seeds, milk, fruits, and several vegetables) was associated with shorter sleep duration. Also, the highest adherence to the dietary pattern named “high fats, refined grains, and meat” (which was defined by high consumption of solid fats, cured meat, potatoes, refined grains, meat, cheese, and added sugars) was associated with an increased risk for sleep disorders and shorter sleep duration [84]. Based on other data from the NHANES, it was also found that there is a reverse U-shaped association between diet quality and sleep duration [85]. Participants with five or fewer hours of sleep at night and those with nine or more hours of sleep at night had poorer overall diet quality (measured with the Healthy Eating Index-2015). Taking into account the individual food groups among participants with short sleep duration, a lower intake of total fruits, whole fruits, total vegetables, greens, beans, whole grains, dairy, total protein, and seafood was observed. On the other hand, among participants with long sleep duration, a higher intake of refined grains and added sugar was observed, but there was also higher consumption of some healthier ingredients (dairy and total protein) [85]. It is also worth noting that plant-based dietary patterns, such as the MD and the DASH diet, are associated with lower levels of inflammatory markers and oxidative stress [86,87,88,89,90], which may have a significant association with sleep quality. It is well known that the basis of the MD and the DASH diet is consumption of vegetables, fruits, and whole grain products, as well as limitation of red meat, sources of saturated fats, and processed foods [15,49,50]. These assumptions make these diets abundant in vitamin C, carotenoids, polyphenols, and minerals, all of which possess anti-inflammatory and/or antioxidant properties [91,92]. Interestingly, plant foods contain some amounts of melatonin and its precursor. Melatonin is a neurohormone involved in the regulation of sleep patterns and circadian rhythms, but it also has anti-inflammatory and antioxidant properties [93]. Zhang et al. [92] showed that the oxidative balance score, which includes dietary and lifestyle factors (16 and 4, respectively), was correlated with sleep quality. Moreover, Hepsomali and Groeger [94] described the role of systemic chronic inflammation in diet health and sleep quality. They suggested that reducing systemic chronic inflammation via dietary interventions could be an effective primary and/or complementary strategy to enhance the quality of sleep. Furthermore, Wang et al. [95] observed the role of dietary inflammation in the relationship between sleep quality and the risk of cardiovascular disease. According to the results of this study, a reduction in inflammatory dietary sources could decrease the effect of sleep disorders on CVD risk. Other studies conducted in different populations have also confirmed that there is an association between the inflammatory potential of the diet and sleep quality [96,97]. Moreover, an association between proinflammatory diets and sleep duration was also found. Kase et al. [98] observed that compared to the participants in the lowest quintile of the energy-adjusted Dietary Inflammatory Index (the most anti-inflammatory diets), participants in the fourth and fifth quintiles (the most proinflammatory diets) had 26% and 40% higher odds of short sleep duration (≤6 h), respectively. Similar associations were also found for long sleep duration (≥9 h). Participants in the fourth and fifth quintiles, compared to those in the first quintile, had 24% and 23% higher odds of long sleep duration, respectively. It was also observed that participants in the highest quintile of the energy-adjusted Dietary Inflammatory Index were more likely to declare sleep disturbances compared to those from the first quintile (OR = 1.14, 95% CI 1.02–1.27). 

Most of the studies presented in this review showed that there is a favorable association between the Mediterranean, DASH, and MIND diets and different sleep parameters. However, there are some limitations that should be taken into account when drawing conclusions, especially the types of these studies. Most of the studies published so far have a cross-sectional character. As exposure and outcome occur at the same time, this type of research does not allow for stating a causal relationship. Cohort studies have a higher strength of evidence. These studies, through long-term observation, examine exposure to a given factor and its impact on health and, therefore, may determine the duration of exposure and outcome. In cohort studies, evidence for causality may be defined. However, both of these types of studies are observational, and nutritional data are collected based on different dietary interviews, which can also have some limitations. Therefore, it would be desirable to conduct more RCTs on this topic, as RCTs allow for the establishment of causality, not only an association between two parameters. What is highly important is that RCTs assess the effectiveness of specific interventions in both a study and control group, with participants randomly assigned to one or the other [99]. The intervention is precisely defined, which in nutritional studies means that the study and control groups receive either an intervention or control diet, with both having precisely defined compositions. A very good example of such a study is the DASH trial [49,50]. Furthermore, the random assignment of participants to study and control groups may cause an equal distribution of confounding variables within those groups [99]. Another limitation that should be mentioned is that some of the presented studies were conducted with a small number of participants and often only among women, which should also be taken into account when designing further studies.

## 7. Conclusions

The vast majority of studies have shown a beneficial association between the MD, the DASH diet, and the MIND diet and sleep quality and/or duration. However, most of the findings were obtained in cross-sectional analysis, and therefore, causality cannot be identified, which was usually emphasized by the authors themselves. There are very limited data on the association between the MIND diet and sleep, but taking into account the results observed so far, this may be a future direction for further research. 

Taking into account the significant role of adequate sleep quality in various diseases and even in all-cause mortality, it seems necessary to include variables related to sleep hygiene, quality, and duration in prospective cohort studies, which would enable an analysis of the associations between these parameters and diet. Furthermore, new RCTs could provide further evidence to confirm the associations observed in existing studies. Nevertheless, the results obtained so far in various populations strongly suggest that healthy dietary patterns, abundant in plant foods, are associated with better sleep quality and more adequate sleep duration. Based on the literature review, it can be posited that the favorable effect of these diets was caused by the synergistic effects of healthy, mainly plant-based, foods. These diets provide an intake of antioxidant and anti-inflammatory compounds, which were observed to be associated with the quality of sleep.

## Figures and Tables

**Table 1:** Summary of the main results according to the relationship between the Mediterranean diet (MD) and sleep quality and/or duration.

Reference/ Type of Study (A, B, C)	Study Group	Main Findings According to the MD
Castro-Diehl et al. [24]/*	*n* = 2068 (different *n* in selected analysis depending on data availability); F and M adults aged 45–84 years were recruited to Exam 1 (2000–2002) of MESA	MD(+) adequate sleep duration and fewer insomnia symptoms. Participants with higher MDA were less likely to have insomnia accompanied by short sleep.
Gupta et al. [25]/(A)	*n* = 2169; F and M	Short sleep duration was associated with lower MDA (F). Inconsistent weekday–weekend sleep duration was related to lower MDA (F and M).
Şahin-Bodur et al. [26]/(A)	*n* = 1031; F and M age 19–64 y.o.	MD(+) better sleep quality. In the low-MDA group, sleep duration was lower than in the moderate- and high-MDA groups. There was an association between wake-up time and MDA groups, but not with bedtime.
Zuraikat et al. [27]/(B)	*n* = 432; F age 20–76 y.o.	Higher MDA at baseline was associated with better sleep quality, higher sleep efficiency, and fewer sleep disturbances at 1-year follow-up.
Godos et al. [28]/(A)	*n* = 1936; F and M age ≥ 18 y.o.	MD(+) better sleep quality in the overall study group and in the normal/overweight participants. In the overall study group, the MD was associated with having adequate sleep quality, sleep duration, latency, and efficiency, with no day dysfunction due to sleepiness. In the normal/overweight participants the MD was associated with having adequate sleep quality, sleep duration, and latency. There were no associations in the obese participants.
Muscogiuri et al. [29]/(A)	*n* = 172; F and M age 51.8 ± 15.6 y.o.	MD(+) better sleep quality.
Huang et al. [30]/(A)	*n* = 7987; F and M age 20–74 y.o.	MD(+) lower poor sleep prevalence.
Shiraseb et al. [31]/(A)	*n* = 266; F age 18–48 y.o.	MD(+) good sleep quality.
Zaidalkilani et al. [32]/(A)	*n* = 917; F age 36 ± 10 y.o.	MD(+) better sleep and fewer insomnia symptoms.
Bakırhan et al. [33]/(A)	*n* = 250; F and M age 19–64 y.o.	MD(+) better sleep quality.
Mantzorou et al. [34]/(A)	*n* = 3254; F and M age ≥ 65 y.o.	MD(+) adequate sleep quality.
Campanini et al. [35]/(B)	*n* = 1596; F and M age ≥ 60 y.o.	Higher MDA was associated with a lower risk of having two or more indicators of poor sleep quality and with a lower risk of large changes in sleep duration.
Mamalaki et al. [36]/(A)	*n* = 1639; F and M age ≥ 65 y.o.	MD(+) sleep quality but not with sleep duration. Sleep quality was positively associated with MDA in participants aged ≤ 75 years, but not in those aged > 75 years.
Buja et al. [37]/(A)	*n* = 267; F and M age 6–7 y.o.	Association between MDA and sleep duration.
Zapata-Lamana et al. [38]/(A)	*n* = 265; F and M age 11–18 y.o.	Having poor sleep hygiene (difficulties in awakening and falling asleep, the presence of nightmares, and morning tiredness) was associated with lower MDA.
Yaghtin et al. [39]/(A)	*n* = 733; F age 12–18 y.o.	MD(+) lower insomnia level.
Rosi et al. [40]/(A)	*n* = 409; F and M age 11–14 y.o.	MD(+) sleep habits (duration, quality, daytime sleepiness).
López-Gil et al. [41]/(A)	*n* = 847; F and M age 12–17 y.o.	Higher MDA was associated with higher odds of meeting sleep recommendations and lower odds of reporting sleep-related problems.
Fernández-Medina et al. [42]/(A)	*n* = 334; F and M age 21.8 ± 6.2 y.o.	Correlation between poor sleep quality and reduced MDA.
Naja et al. [43]/(A)	*n* = 503; F and M age 22.1 ± 4.2	MD(+) morningness chronotype, improved overall sleep quality and sleep components (less sleep latency, sleep disturbance, daytime dysfunction). MD(−) sleep duration, sleep efficiency, and the use of sleep medications.
Mahmoudzadeh et al. [44]/(A)	*n* = 181; F age 18–25 y.o.	MD(+) lower degree of insomnia and daytime sleepiness.
Flor-Alemany et al. [45]/ (A)	*n* = 150; pregnant F at the 16th g.w. age 32.9 ± 4.6 y.o.	MD(+) better sleep quality along the pregnancy course.
Casas et al. [46]/(C)	*n* = 680; pregnant F (enrolment 19–23.6th g.w.; end of intervention 34–36th g.w.)	MD intervention improved sleep quality during gestation.
Verkaar et al. [47]/ (A, B)	*n* = 2629 dietary data (different *n* for cross-sectional and longitudinal analyses and with objective and subjective sleep data); F and M	No significant associations between DPs (MD, among others) and total sleep time, sleep onset latency, wake after sleep onset, sleep efficiency and subjective sleep quality.
van Egmond et al. [48]/(A)	*n* = 970; M age 71.0 ± 0.6 y.o.	MD(−) sleep initiating, sleep maintenance problems.

MD—Mediterranean diet; (A)—cross-sectional study; (B)—prospective cohort study, longitudinal study; (C)—randomized clinical trial; *n*—number of participants; F—female; M—male; y.o.—years old; MD(+) means that the MD was associated with the parameters shown in Table; MD(−) means that there was no association between the MD and the parameters shown in Table; MDA—Mediterranean diet adherence; *—cross-sectional analysis of the association between the alternate Mediterranean diet (aMed) score and sleep duration at Exam 5 (2010–2012) of MESA, supplemented with analyses of the associations between sleep measures and changes in the aMed score from Exam 1 (2000–2002) to Exam 5; MESA—Multi-Ethnic Study of Atherosclerosis; g.w.—gestational week; DPs—dietary patterns.

**Table 2:** Summary of the main results according to the relationship between the DASH diet and sleep quality and/or duration.

Reference/ Type of Study (A, B, C)	Study Group	Main Findings According to the DASH Diet
Huang et al. [30]/(A)	*n* = 7987; F and M age 20–74 y.o.	DASH diet (+) lower poor sleep prevalence.
Shiraseb et al. [31]/(A)	*n* = 266; F age 18–48 y.o.	DASH diet (+) good sleep quality and circadian rhythm.
Daneshzad et al. [63]/(B)	*n* = 66; F (diabetes) age in the DASH diet group: 57.52 ± 4.99 y.o.; age in the control group: 60.70 ± 6.33 y.o.	Sleep quality and night sleep duration significantly increased over 12 weeks in the DASH diet group.
Liang et al. [64]/(A)	*n* = 3941; F and M age ≥ 30 y.o.	DASH diet (+) lower poor sleep-related daytime dysfunction. DASH diet (−) sleepiness and sleep disturbance.
Saharkhiz et al. [65]/(A)	*n* = 181; F age 18–25 y.o.	DASH diet (+) lower difficulty with sleep initiation. DASH diet (−) insomnia, daytime sleepiness, short sleep duration.
Rostami et al. [66]/(A)	*n* = 488; F age 12–18 y.o.	DASH diet (+) lower insomnia.
Pahlavani et al. [67]/(A)	*n* = 535; F age 12–18 y.o.	DASH diet (+) lower daytime sleepiness score.
Karbasi et al. [68]/(A)	*n* = 350 breastfeeding mothers and their infants age 29.5 ± 5.9 y.o.	DASH diet (+) lower score of sleep disorders and shorter sleep latency in mothers, higher mother sleep efficiency, lower score of sleep disorders in infants. DASH diet (−) need for sleep medications and daytime dysfunction among mothers.
Khorasanchi et al. [69]/(C)	*n* = 246 (123 cases of recovered COVID-19 patients and 123 controls without a history of COVID-19); F and M age ≥ 30 y.o.	DASH diet (−) sleep quality or insomnia.

DASH—Dietary Approaches to Stop Hypertension; (A)—cross-sectional study; (B)—randomized controlled trial; (C)—case-control study; *n*—number of participants; F—female; M—male; y.o.—years old; DASH diet (+) means that the DASH diet was associated with the parameters shown in the Table; DASH diet (−) means that there was no association between the DASH diet and the parameters shown in the Table.

**Table 3:** Summary of the main results according to the relationship between the MIND diet and sleep quality and/or duration.

Reference/ Type of Study	Study Group	Main Findings According to the MIND Diet
Rostami et al. [82]/(A)	*n* = 400; M age 38.67 ± 5.26 y.o.	Higher adherence to the MIND diet was associated with lower odds of poor sleep quality, daytime sleepiness, and insomnia.
Fox et al. [83]/(A)	*n* = 1205; F and M with Parkinson’s disease age 36–90 y.o.	The MIND diet score was associated with a reduction in all non-motor symptoms assessed in the study (constipation, motivation, depression, withdrawal, anxiety, fatigue, daytime sleepiness, visual disturbance, insomnia, REM sleep behavior disorder, muscle pain, forgetfulness, comprehension, sexual dysfunction, urinary symptoms, hallucinations).

MIND—Mediterranean–DASH Intervention for Neurodegenerative Delay; (A)—cross-sectional study; *n*—number of participants; F—female; M—male; y.o.—years old.
